# Supplementary material for: LC3-associated phagocytosis promotes glial degradation of axon debris after injury in Drosophila models
Source: Nat Commun. 2023 May 29;14:3077. doi: 10.1038/s41467-023-38755-4 (PMC10227080; doi:10.1038/s41467-023-38755-4)
Supplement: Supplementary file 1 — Supplementary Information [file 41467_2023_38755_MOESM1_ESM.pdf]

Supplementary Materials for

LC3-associated phagocytosis promotes glial degradation of axon debris after injury in *Drosophila* models

Áron Szabó[1]*, Virág Vincze[1#], Aishwarya Sanjay Chhatre[1,2#], András Jipa[1], Sarolta Bognár[1], Katalin Eszter Varga[1], Poulami Banik[1], Adél Harmatos-Ürmösi[1], Lukas J. Neukomm[3] and Gábor Juhász[1,4]*

*Corresponding authors. Email: aszabo@brc.hu, juhasz.gabor@brc.hu

This file includes:

Supplementary Figures 1 to 10

Supplementary Table 1.

a

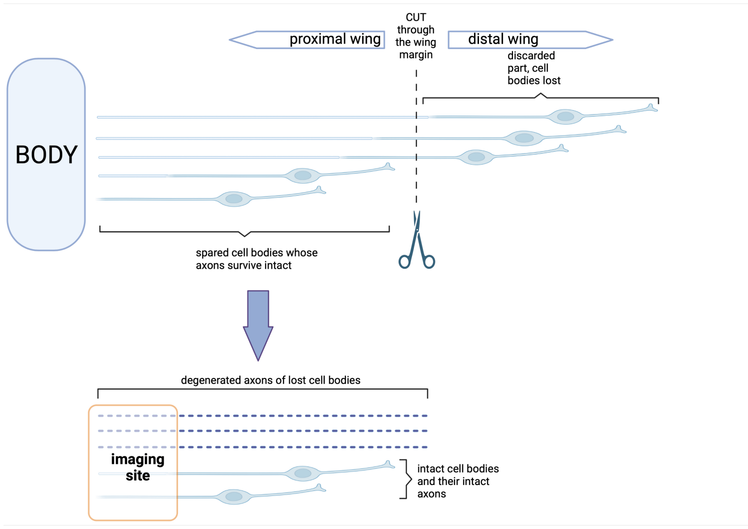

proximal wing
CUT through the wing margin
distal wing
discarded part, cell bodies lost
BODY
spared cell bodies whose axons survive intact
degenerated axons of lost cell bodies
imaging site
intact cell bodies and their intact axons

b

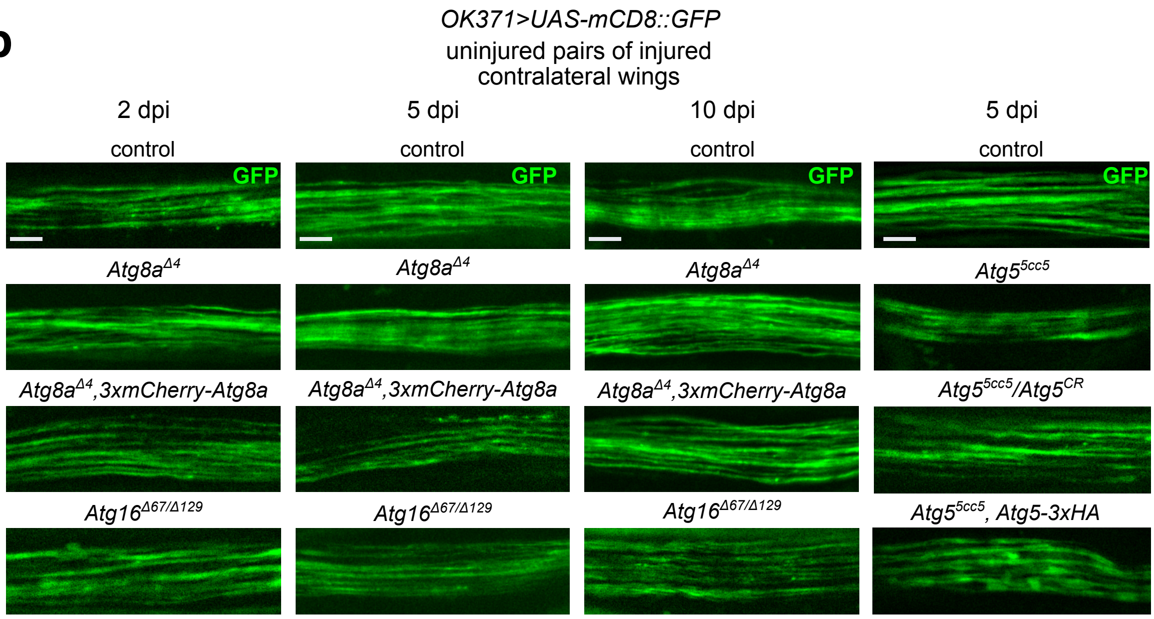

OK371>UAS-mCD8::GFP
uninjured pairs of injured contralateral wings
2 dpi
5 dpi
10 dpi
5 dpi
control
control
control
control
GFP
GFP
GFP
GFP
Atg8aΔ4
Atg8aΔ4
Atg8aΔ4
Atg5 5cc5
Atg8aΔ4,3xmCherry-Atg8a
Atg8aΔ4,3xmCherry-Atg8a
Atg8aΔ4,3xmCherry-Atg8a
Atg5 5cc5/Atg5 CR
Atg16 Δ67/Δ129
Atg16 Δ67/Δ129
Atg16 Δ67/Δ129
Atg5 5cc5, Atg5-3xHA

c

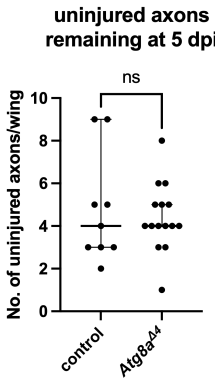

uninjured axons remaining at 5 dpi
ns
No. of uninjured axons/wing
0
2
4
6
8
10
control
Atg8aΔ4

Supplementary Figure 1. Schematic of the wing injury model and uninjured contralateral wing nerves for genotypes shown in Figure 1

a Injury paradigm of the L1 vein wing nerve. After wing transection, Wallerian degeneration results in debris generation proximal to the fly body from the injury site in case of axons that are cut. The distal half of the wing is discarded. Neurons in the proximal wing portion are unaffected so their projecting axons remain uninjured. **b** Single slice images of uninjured wing nerve segments (pairs of contralateral injured wings presented in Fig. 1b and d) are shown for the indicated genotypes on *OK371> UAS-mCD8::GFP/+* background at 2, 5 and 10 days post contralateral wing injury. For the $Atg5^{5cc5}$/ $Atg5^{CR00038}$ genotype, female flies were used. **c** Quantification of remaining uninjured axons in injured wing nerves of the indicated genotypes at 5 dpi from Fig 1b. Statistical analysis was performed with unpaired, two-tailed Mann-Whitney test. The graph shows the median with 95% confidence intervals. n=9, 15 biologically independent animals. p=0.8493, ns - not significant. Scale bar: 5 µm. Source data are provided as a Source Data file.

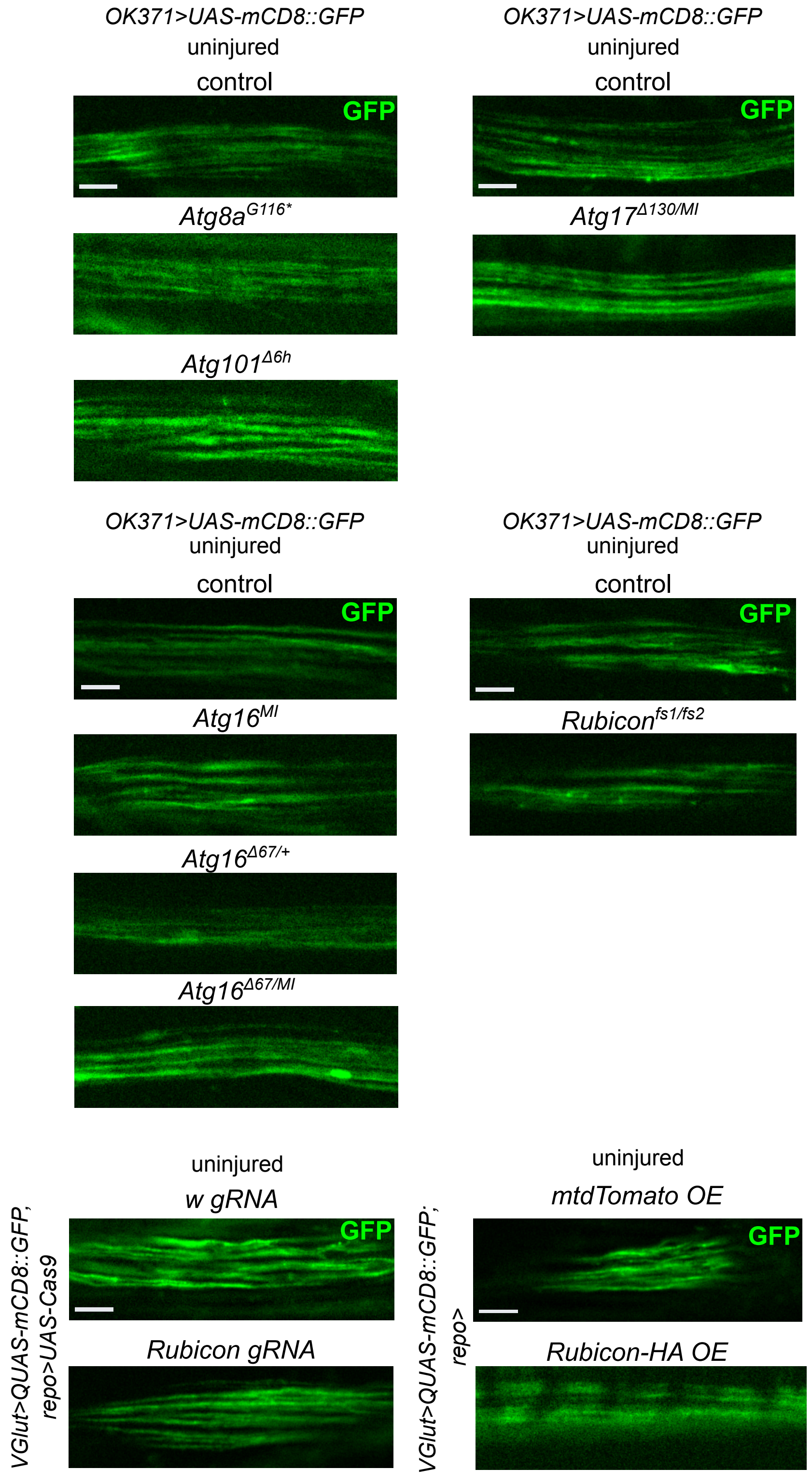
OK371>UAS-mCD8::GFP
uninjured
control
GFP
$Atg8a^{G116*}$
$Atg101^{\Delta 6h}$
OK371>UAS-mCD8::GFP
uninjured
control
GFP
$Atg17^{\Delta 130/MI}$
OK371>UAS-mCD8::GFP
uninjured
control
GFP
$Atg16^{MI}$
$Atg16^{\Delta 67/+}$
$Atg16^{\Delta 67/MI}$
OK371>UAS-mCD8::GFP
uninjured
control
GFP
$Rubicon^{fs1/fs2}$
VGlut>QUAS-mCD8::GFP,
repo>UAS-Cas9
uninjured
w gRNA
GFP
Rubicon gRNA
VGlut>QUAS-mCD8::GFP;
repo>
uninjured
mtdTomato OE
GFP
Rubicon-HA OE

Supplementary Figure 2. Uninjured contralateral wing nerves for genotypes in Figures 2 and 6

Single slice images of uninjured wing nerves of the indicated genotypes on *OK371> UAS-mCD8::GFP/+* or *VGlut> QUAS-mCD8::GFP/+* background corresponding to Fig. 2a, b, and f and Fig. 6g, i, and k. For the *Rubicon*^{fs1}/*Rubicon*^{fs2} genotype, female flies were used. Injured counterparts are presented in Figs. 2 and 6. Scale bar: 5 μm. Experiments were independently repeated twice with similar results.

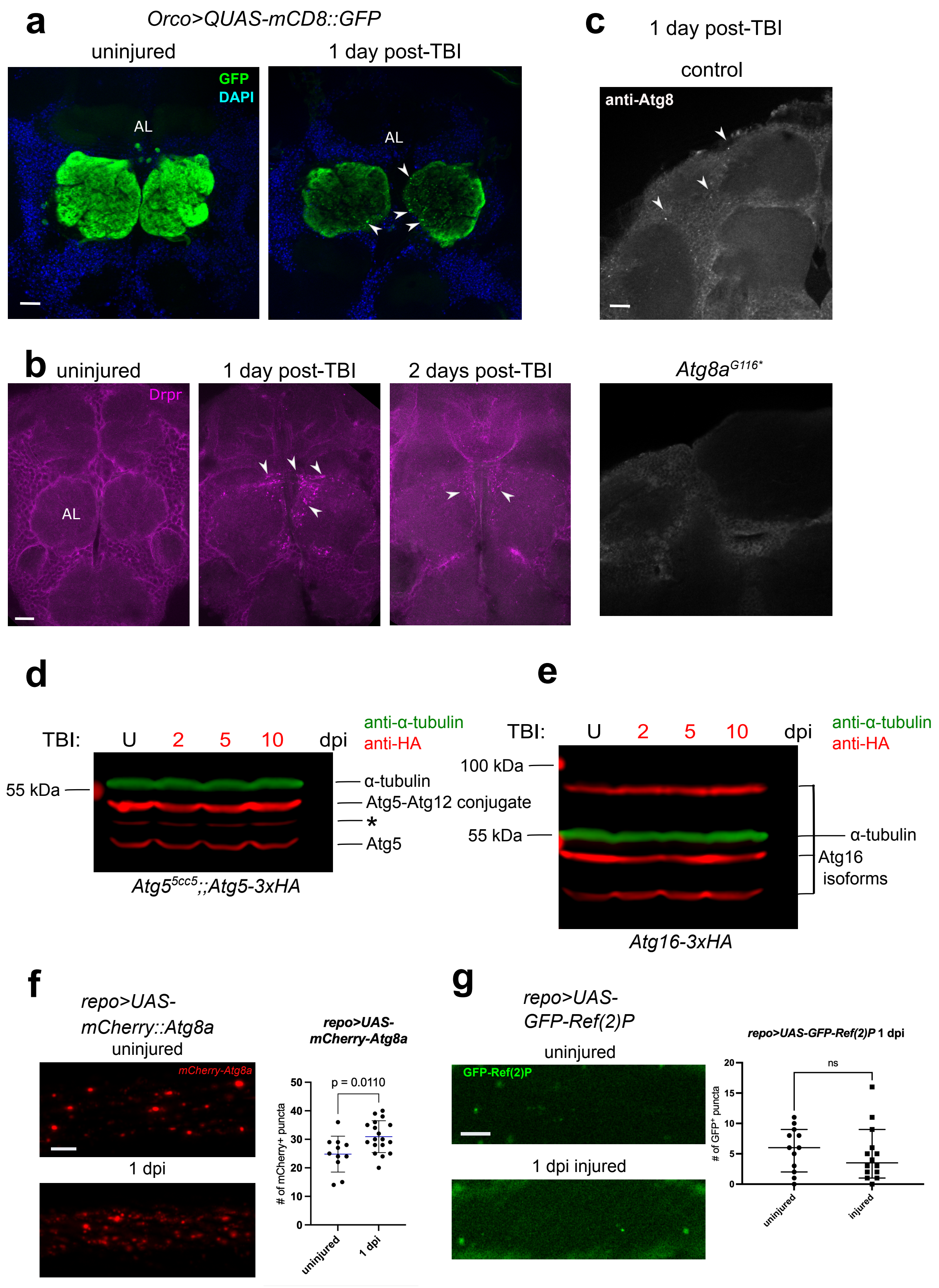
a
Orco>QUAS-mCD8::GFP
uninjured
1 day post-TBI
GFP
DAPI
AL
AL
c
1 day post-TBI
control
anti-Atg8
b
uninjured
1 day post-TBI
2 days post-TBI
Drpr
AL
Atg8a G116*
d
TBI: U 2 5 10 dpi
anti-α-tubulin
anti-HA
55 kDa
α-tubulin
Atg5-Atg12 conjugate
*
Atg5
Atg5 5cc5;;Atg5-3xHA
e
TBI: U 2 5 10 dpi
anti-α-tubulin
anti-HA
100 kDa
55 kDa
α-tubulin
Atg16 isoforms
Atg16-3xHA
f
repo>UAS-mCherry::Atg8a
uninjured
mCherry-Atg8a
1 dpi
repo>UAS-mCherry-Atg8a
p = 0.0110
# of mCherry+ puncta
uninjured
1 dpi
g
repo>UAS-GFP-Ref(2)P
uninjured
GFP-Ref(2)P
1 dpi injured
repo>UAS-GFP-Ref(2)P 1 dpi
ns
# of GFP+ puncta
uninjured
injured

Supplementary Figure 3. Additional traumatic brain injury (TBI) and wing injury data

a *Orco> QUAS-mCD8::GFP* adults 1-3 days post eclosion were subjected to a single round of TBI as described in Methods. Brains were stained for GFP to visualize axons in the antennal lobe (AL) in the central brain of uninjured and injured flies at 1 dpi. TBI leads to fragmentation of axons evidenced by appearance of bead-like structures (arrowheads) along axonal projections in the AL. Scale bar: 20 µm. **b** Control 1-3 days old *w* adults were subjected to a single round of TBI. Brains were stained for Drpr at the indicated time points to reveal phagocytic glia and forming phagosomes in glia (arrowheads). Scale bar: 20 µm. **c** Control *w* and $Atg8a^{G116*}$ 1-3 days old adults were subjected to TBI. Endogenous Atg8a puncta form in control but not in $Atg8a^{G116*}$ brains at 1 dpi.). Scale bar: 20 µm. **d, e** Western blots of $Atg5^{5cc5}$, *Atg5-3xHA* (**d**) and *Atg16-3xHA* (**e**) head extracts from uninjured (U) and TBI treated flies collected at 2, 5 and 10 days post-TBI (dpi). Atg5 and Atg16 forms are detected by anti-HA (red) and anti-α-tubulin (green) serves as loading control. Asterisk: non-specific band. **f** Left: *repo>mCherry-Atg8a* wing nerve images. Scale bar: 5 µm. Right: quantification of the number of mCherry-Atg8a puncta in single-slice images of *repo>mCherry-Atg8a* in uninjured and injured wings. Unpaired, two-tailed t-test, n=11, 18 biologically independent animals. **g** Left: uninjured and injured *repo>UAS-GFP-ref(2)P* wings. Scale bar: 5 µm. Right: quantification of the number of GFP-Ref(2)P^{+} puncta in single-slice images of *repo>UAS-GFP-ref(2)P*. Unpaired, two-tailed Mann-Whitney test n=12, 14 biologically independent animals. p= 0.4079, ns - not significant. **a**-**g**. Experiments were independently repeated twice with similar results. Source data are provided as a Source Data file.

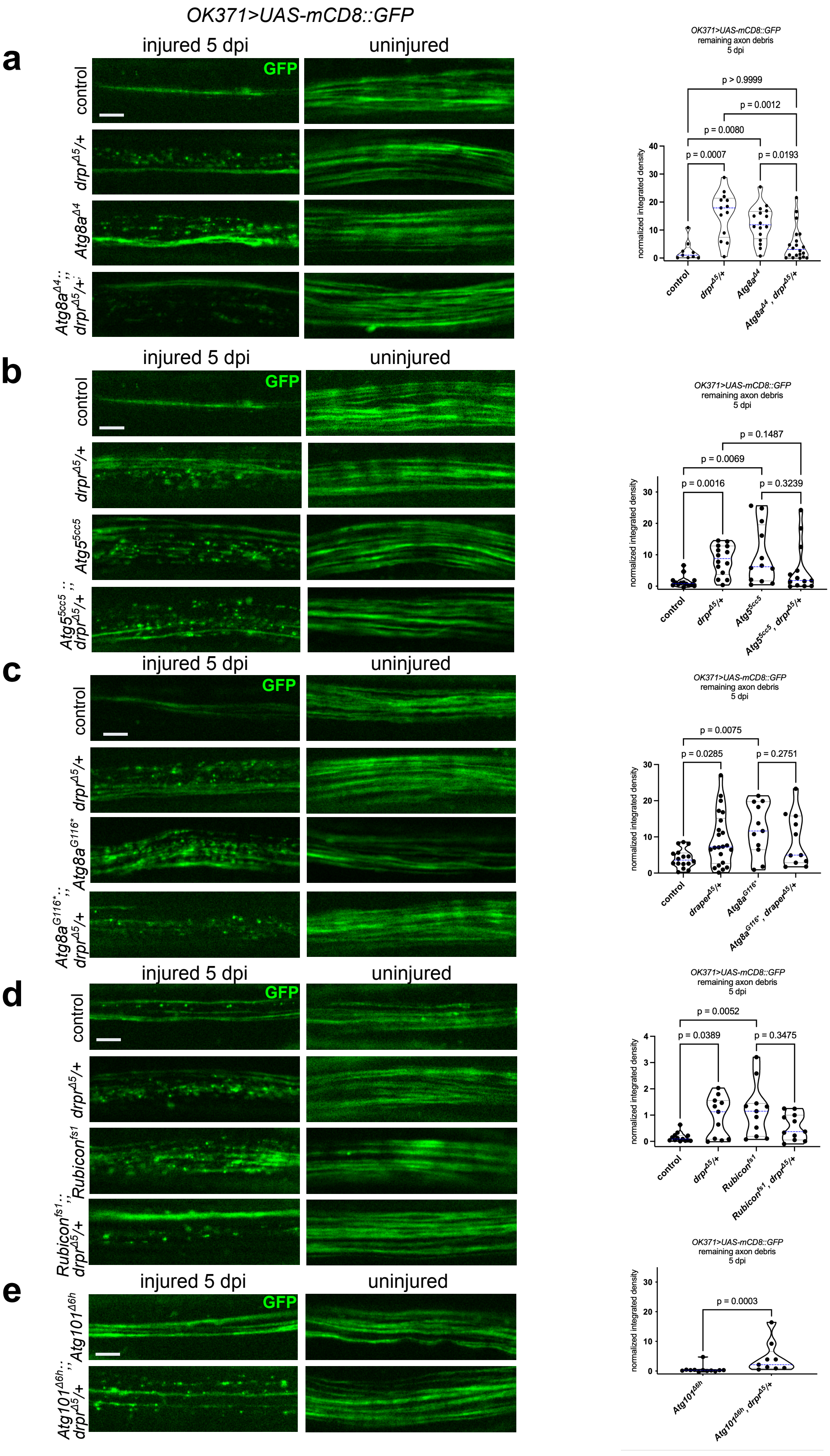
OK371>UAS-mCD8::GFP
a
injured 5 dpi
uninjured
GFP
control
drprΔ5/+
Atg8aΔ4
Atg8aΔ4;; drprΔ5/+
OK371>UAS-mCD8::GFP remaining axon debris 5 dpi
p > 0.9999
p = 0.0012
p = 0.0080
p = 0.0007
p = 0.0193
normalized integrated density
b
Atg5^5cc5
Atg5^5cc5;; drprΔ5/+
p = 0.1487
p = 0.0069
p = 0.0016
p = 0.3239
c
Atg8a^G116*
Atg8a^G116*;; drprΔ5/+
p = 0.0075
p = 0.0285
p = 0.2751
draperΔ5/+
Atg8a^G116*, draperΔ5/+
d
Rubicon^fs1
Rubicon^fs1;; drprΔ5/+
p = 0.0052
p = 0.0389
p = 0.3475
e
Atg101^Δ6h
Atg101^Δ6h;; drprΔ5/+
p = 0.0003

Supplementary Figure 4. Genetic interaction tests between mutations for genes encoding Atg8a conjugation machinery members, Rubicon and Atg101 with a heterozygous *drpr* mutation

a-**e** Left: Single optical slices of degenerating and contralateral uninjured wing nerves of the indicated genotypes on *OK371> UAS-mCD8::GFP/+* background at 5 dpi. Scale bar: 5 μm. **a**-**e** Right: Quantification of axon debris in single-slice images of the indicated genotypes at 5 dpi. **a** Kruskal-Wallis test with Dunn's multiple comparisons test. $p<0.0001$. n=9, 13, 16, 20 biologically independent animals. **b** Kruskal-Wallis test with Dunn's multiple comparisons test. p=0.0011. n=14, 14, 12, 13 biologically independent animals. **c** One-way ANOVA with Holm-Šídák's multiple comparisons test, p=0.0149. n=16, 22, 11, 11 biologically independent animals. **d** Kruskal-Wallis test with Dunn's multiple comparisons test. p=0.0102. n=13, 11, 11, 11 biologically independent animals. **e** Unpaired, two-tailed Mann-Whitney test was used for statistics. n=12 and 9 biologically independent animals. Truncated violin plots with median and quartiles are shown. p values indicated in panels **a**-**d** are corrected for multiple comparisons. Source data are provided as a Source Data file.

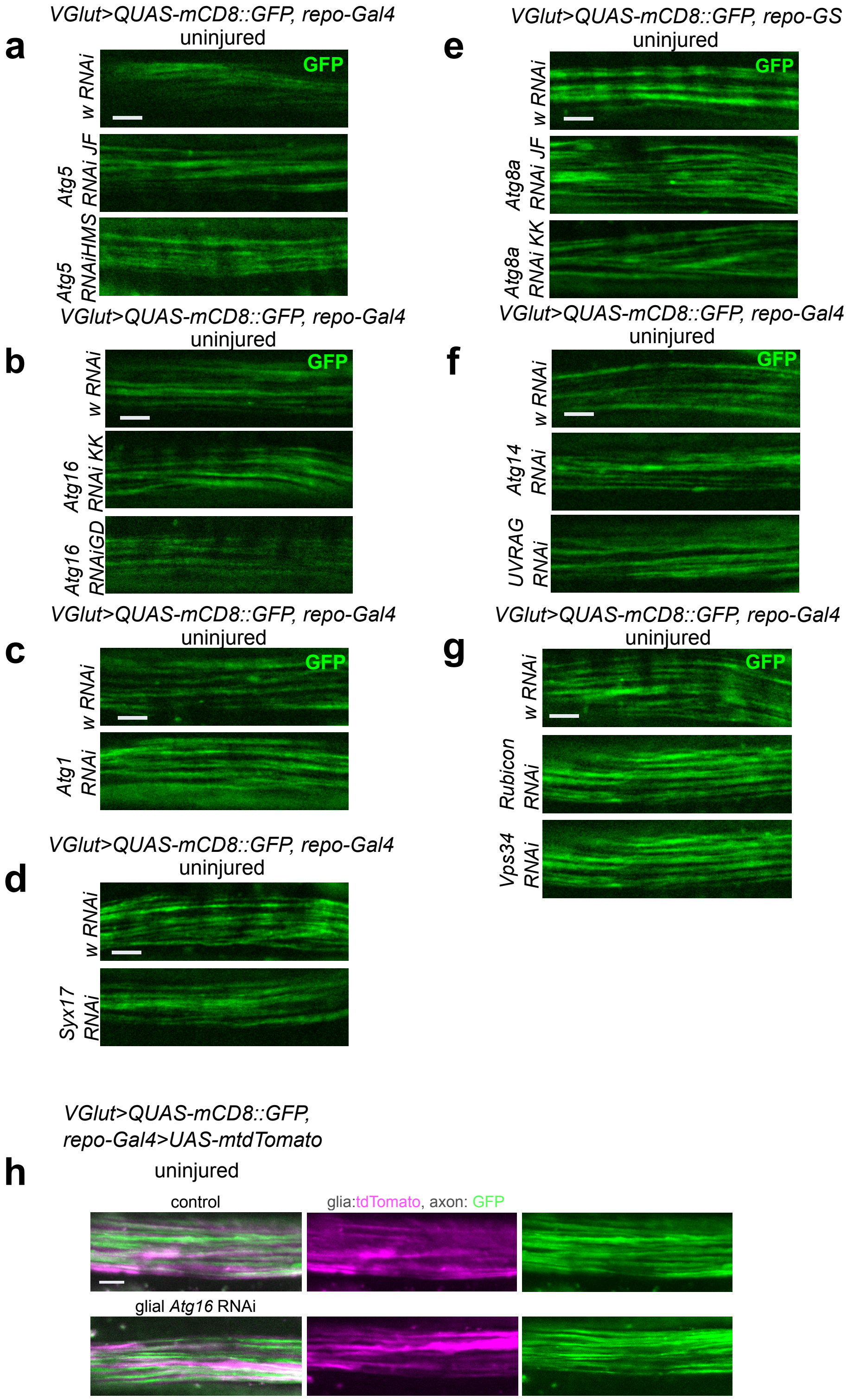

a
VGlut>QUAS-mCD8::GFP, repo-Gal4
uninjured
GFP
w RNAi
Atg5 RNAi JF
Atg5 RNAiHMS
e
VGlut>QUAS-mCD8::GFP, repo-GS
uninjured
GFP
w RNAi
Atg8a RNAi JF
Atg8a RNAi KK
b
VGlut>QUAS-mCD8::GFP, repo-Gal4
uninjured
GFP
w RNAi
Atg16 RNAi KK
Atg16 RNAiGD
f
VGlut>QUAS-mCD8::GFP, repo-Gal4
uninjured
GFP
w RNAi
Atg14 RNAi
UVRAG RNAi
c
VGlut>QUAS-mCD8::GFP, repo-Gal4
uninjured
GFP
w RNAi
Atg1 RNAi
g
VGlut>QUAS-mCD8::GFP, repo-Gal4
uninjured
GFP
w RNAi
Rubicon RNAi
Vps34 RNAi
d
VGlut>QUAS-mCD8::GFP, repo-Gal4
uninjured
w RNAi
Syx17 RNAi
h
VGlut>QUAS-mCD8::GFP,
repo-Gal4>UAS-mtdTomato
uninjured
control
glia:tdTomato, axon: GFP
glial Atg16 RNAi

Supplementary Figure 5. Uninjured contralateral wing nerves of genotypes in Figures 3 and 6, and glial morphology upon *Atg16* knockdown

a-g Single optical slices of uninjured wing nerves expressing the indicated RNAi-s in glia, driven by *repo-Gal4* or *repo- GS* on *VGlut> QUAS-mCD8::GFP/+* background. Injured contralateral wing nerves are presented in Fig. 3a, c, e, i, k and Fig. 6a, c. **h** Single slice images of uninjured wing nerves with or without *Atg16 RNAi KK* in glia driven by *repo-Gal4*, axons labelled with mCD8::GFP, glia labelled with membrane-targeted tdTomato (*VGlut> QUAS-mCD8::GFP, repo-Gal4> UAS-mtdTomato)*. Conclusions were drawn from the observation of n=18 biologically independent animals from each genotype. Scale bar: 5 μm. **a-h** Experiments were independently repeated twice with similar results.

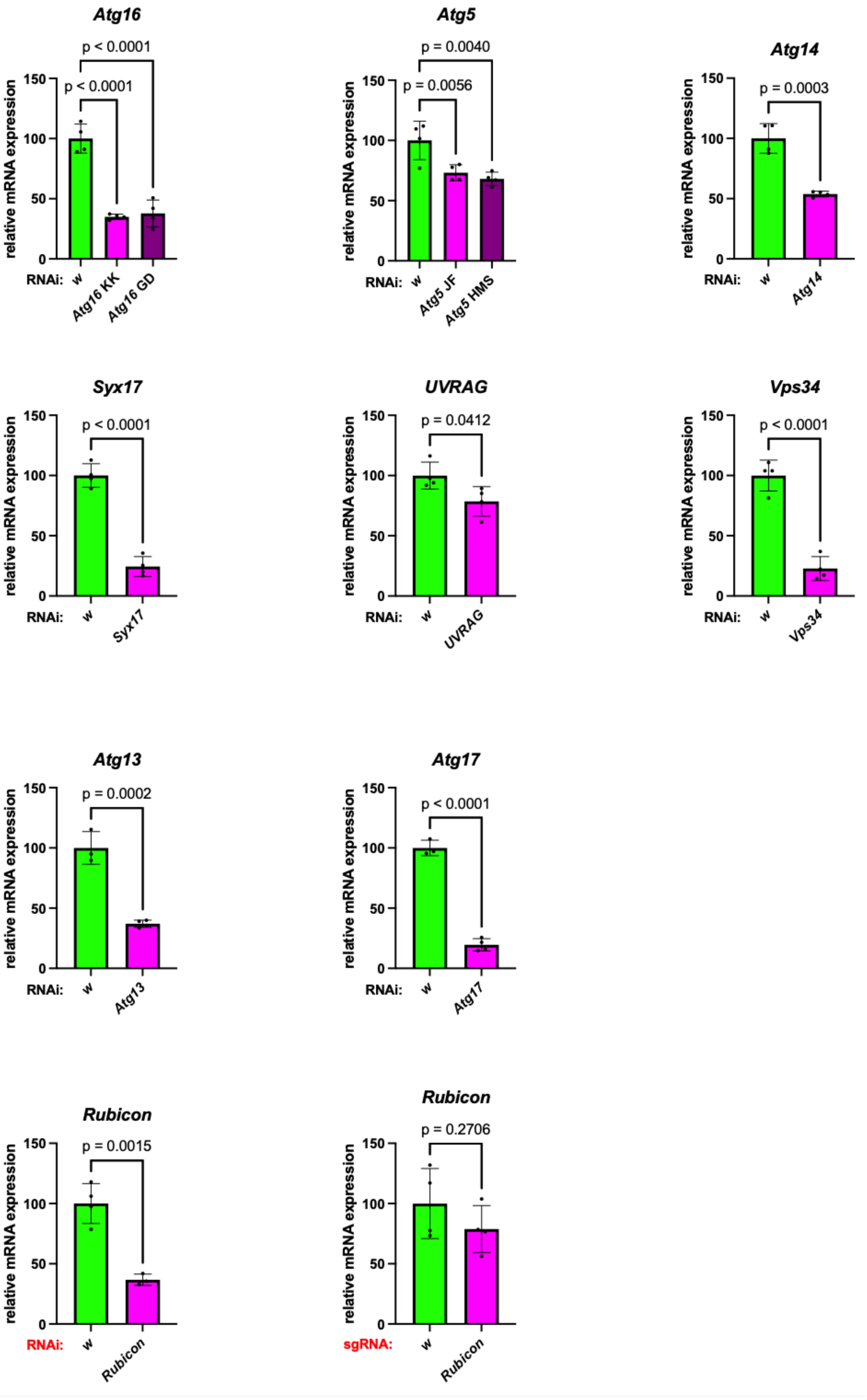

Atg16
p < 0.0001
p < 0.0001
relative mRNA expression
RNAi:
w
Atg16 KK
Atg16 GD
Atg5
p = 0.0040
p = 0.0056
Atg5 JF
Atg5 HMS
Atg14
p = 0.0003
Syx17
p < 0.0001
UVRAG
p = 0.0412
Vps34
p < 0.0001
Atg13
p = 0.0002
Atg17
p < 0.0001
Rubicon
p = 0.0015
Rubicon
p = 0.2706
sgRNA:

Supplementary Figure 6. Validation of RNAi transgene knockdown specificity

mRNA levels of RNAi targets measured by quantitative reverse-transcription-coupled PCR (qRT-PCR) in whole bodies of the indicated genotypes driven by *tubulin-Gal4*. *w* RNAi serves as a negative control. *Rubicon* and *w* gRNA effects with *tubulin-Gal4* and *UAS-Cas9* on *Rubicon* mRNA were also measured. Statistics was performed either with unpaired, two-tailed t-test or with one-way ANOVA with Holm-Šídák's multiple comparisons test. Shown is the mean with deviation. n=3 or 4 biologically independent RNA samples for all, sample sizes are indicated by the number of dots as data points in panels. Source data are provided as a Source Data file.

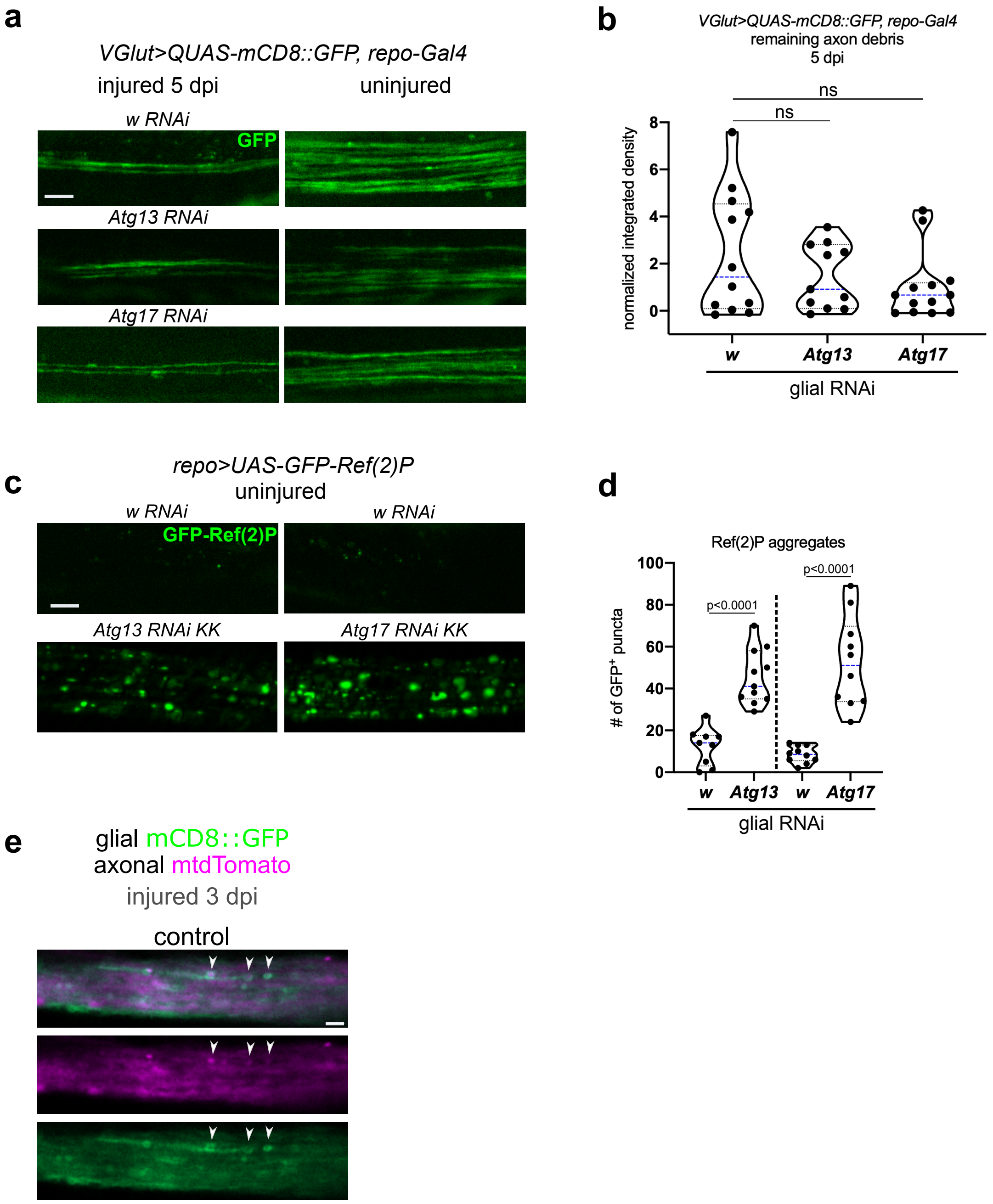
a
VGlut>QUAS-mCD8::GFP, repo-Gal4
injured 5 dpi
uninjured
w RNAi
GFP
Atg13 RNAi
Atg17 RNAi
b
VGlut>QUAS-mCD8::GFP, repo-Gal4
remaining axon debris
5 dpi
ns
ns
normalized integrated density
8
6
4
2
0
w
Atg13
Atg17
glial RNAi
c
repo>UAS-GFP-Ref(2)P
uninjured
w RNAi
w RNAi
GFP-Ref(2)P
Atg13 RNAi KK
Atg17 RNAi KK
d
Ref(2)P aggregates
p<0.0001
p<0.0001
# of GFP+ puncta
100
80
60
40
20
0
w
Atg13
w
Atg17
glial RNAi
e
glial mCD8::GFP
axonal mtdTomato
injured 3 dpi
control

Supplementary Figure 7. The Atg1 kinase complex in glia does not affect debris elimination, and evidence for phagosome formation in wing glia

a Single slice images of degenerating wing nerves expressing the indicated RNAi-s in glia, driven by *repo-Gal4* on *VGlut> QUAS-mCD8::GFP/+* background at 5 dpi, and contralateral uninjured wing nerves. **b** Quantification of axon debris abundance in single-slice images of the indicated genotypes as in (**a**) at 5 dpi, Kruskal-Wallis test. n=12, 13 and 11 biologically independent animals. p=0.2588, 0.6041, respectively, ns - not significant**.** **c** Single-slice images of uninjured wing L1 vein glia expressing *repo-Gal4*-driven *UAS-GFP-ref(2)P* and co-expressing the indicated RNAi-s. Scale bars: 5 μm. **d** Quantification of the number of GFP-Ref(2)P puncta in single-slice images of genotypes in **c**, unpaired, two-tailed t-test. n= 9, 11, 10 and 10 biologically independent animals. Independent experiments are separated by a dashed line. **b, d** Truncated violin plots with median and quartiles are shown. *w* RNAi serves as negative control. **e** Confocal single-slice images of injured wing nerves at 3 dpi, glial membrane is labelled with mCD8::GFP, axons with membrane-targeted tdTomato (*repo-Gal4>UAS-mCD8::GFP, nSyb> lexAop-CD4::tdTomato)*. Arrowheads point to co-localizing puncta. Scale bar: 2 μm. **a-e** Experiments were independently repeated twice with similar results. Source data are provided as a Source Data file.

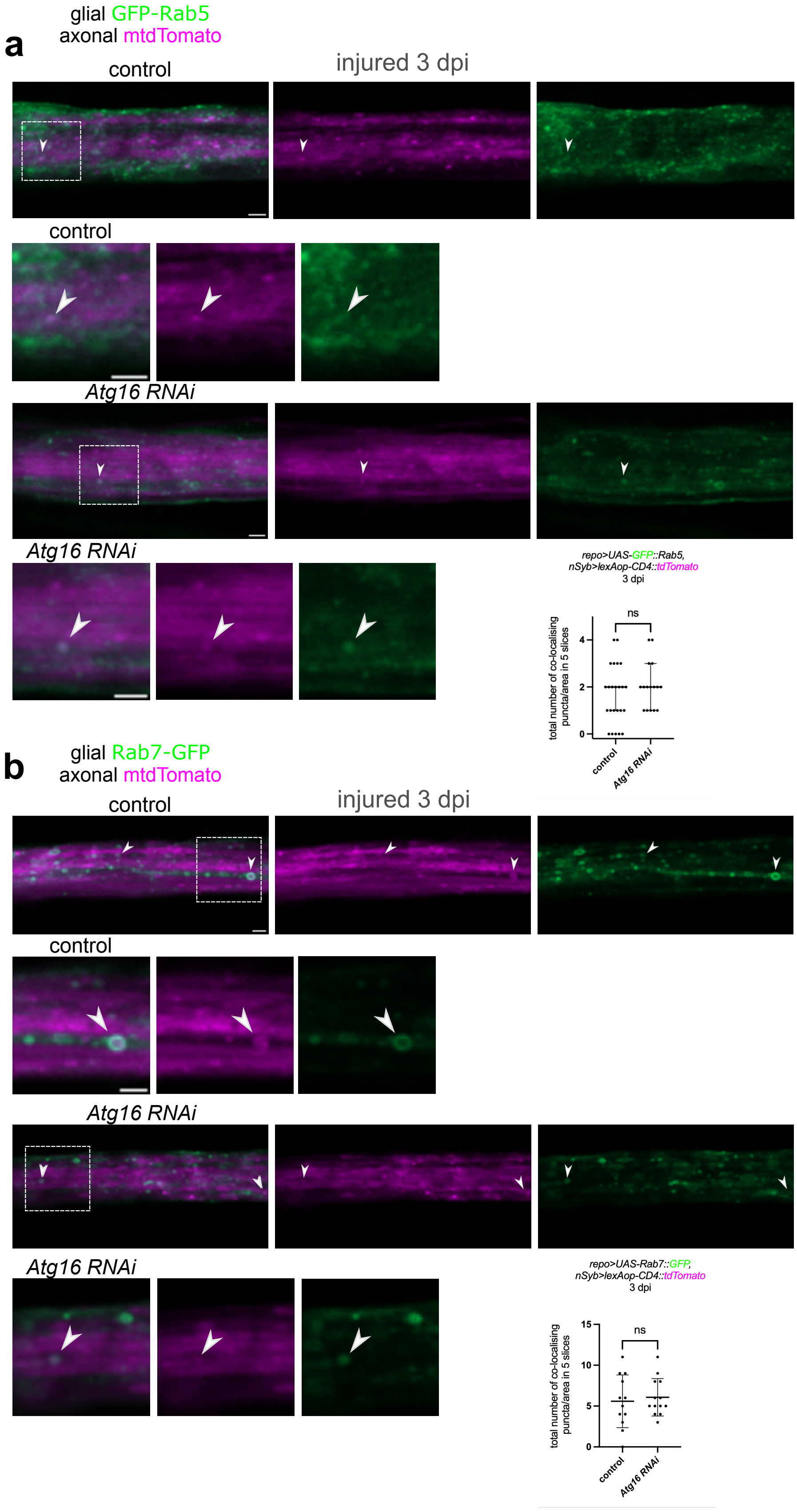
a
glial GFP-Rab5
axonal mtdTomato
control
injured 3 dpi
control
Atg16 RNAi
Atg16 RNAi
repo>UAS-GFP::Rab5,
nSyb>lexAop-CD4::tdTomato
3 dpi
ns
total number of co-localising puncta/area in 5 slices
0
2
4
control
Atg16 RNAi
b
glial Rab7-GFP
axonal mtdTomato
control
injured 3 dpi
control
Atg16 RNAi
Atg16 RNAi
repo>UAS-Rab7::GFP,
nSyb>lexAop-CD4::tdTomato
3 dpi
ns
total number of co-localising puncta/area in 5 slices
0
5
10
15
control
Atg16 RNAi

Supplementary Figure 8. Phagocytic debris processing proceeds normally to the late phagosome stage in the absence of *Atg16*

Confocal single-slice images of injured wing nerves at 3 dpi with or without the expression of *Atg16* RNAi in glia with *repo-Gal4*, glial phagosomes/endosomes are labelled with: **a**: GFP-Rab5 (early phagosome/endosome) or **b**: Rab7-GFP (late phagosome/endosome) and axons with membrane-targeted tdTomato (*repo-Gal4>UAS-GFP-Rab5 <u>or</u> UAS-Rab7-GFP, nSyb> lexAop-CD4::tdTomato)*. Arrowheads point to co-localizing puncta. Magnified images of the area outlined by the dashed rectangle are shown below. Scale bar: 2 μm for all. Bottom graphs: quantification of the number of co-localizing GFP-Rab5/Rab7-GFP and CD4::tdTomato puncta in 5 consecutive single-slice images of genotypes in **a** and **b**. Co-localizing puncta were counted in randomly selected 40 x 12 μm areas. Statistical analysis was performed with unpaired, two-tailed Mann-Whitney test. The graphs show the median with 95% confidence intervals. **a:** n=24, 16 biologically independent animals, p= 0.3171, ns - not significant. **b:** n=12, 13 biologically independent animals, p=0.6616, ns - not significant. Source data are provided as a Source Data file.

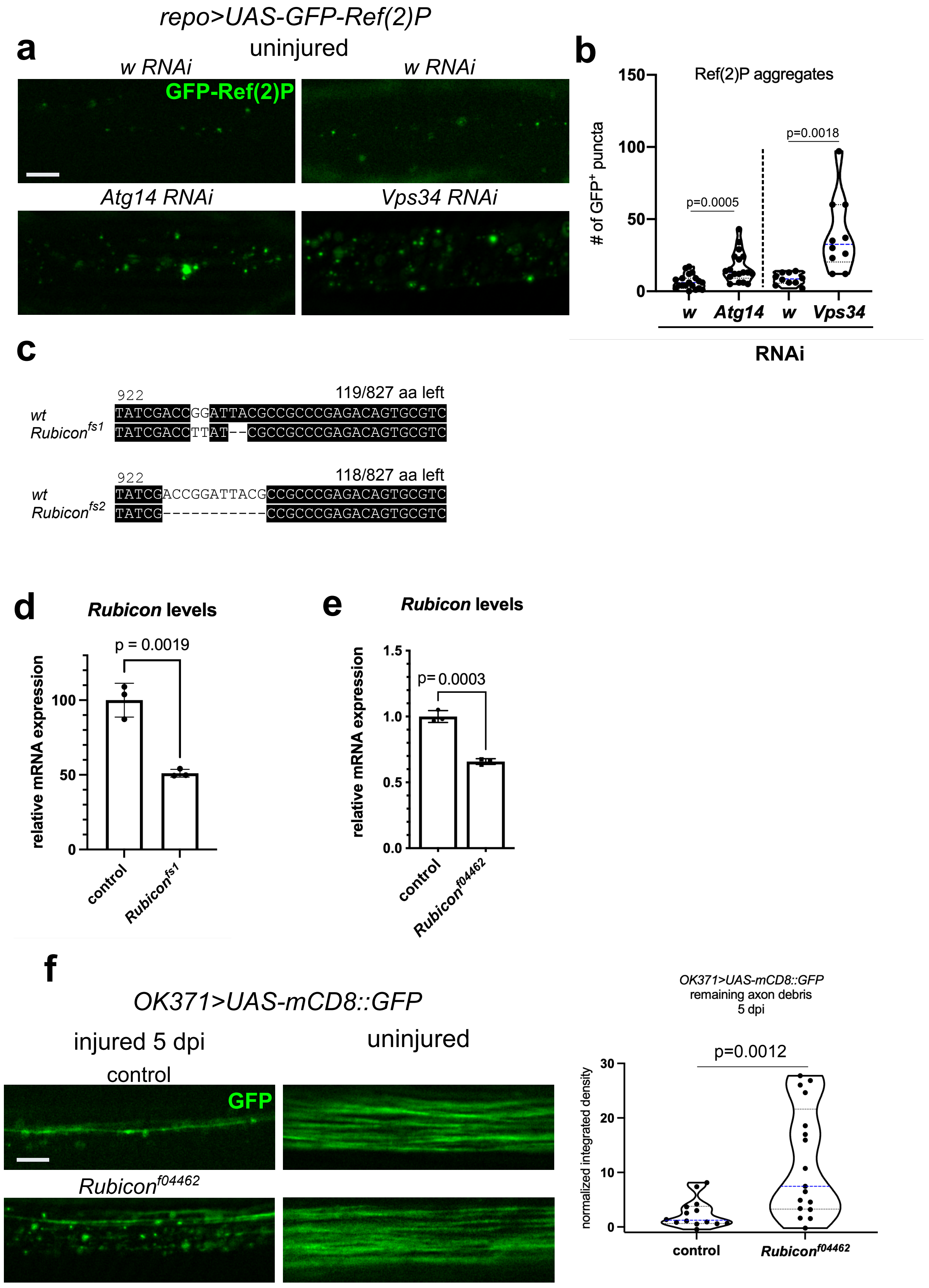
repo>UAS-GFP-Ref(2)P
a
uninjured
w RNAi
w RNAi
GFP-Ref(2)P
Atg14 RNAi
Vps34 RNAi
b
Ref(2)P aggregates
# of GFP+ puncta
150
100
50
0
p=0.0005
p=0.0018
w
Atg14
w
Vps34
RNAi
c
922
119/827 aa left
wt
Rubicon fs1
TATCGACCGGATTACGCCGCCCGAGACAGTGCGTC
TATCGACCTTAT--CGCCGCCCGAGACAGTGCGTC
922
118/827 aa left
wt
Rubicon fs2
TATCGACCGGATTACGCCGCCCGAGACAGTGCGTC
TATCG-----------CCGCCCGAGACAGTGCGTC
d
Rubicon levels
p = 0.0019
relative mRNA expression
100
50
0
control
Rubicon fs1
e
Rubicon levels
p= 0.0003
relative mRNA expression
1.5
1.0
0.5
0.0
control
Rubicon f04462
f
OK371>UAS-mCD8::GFP
injured 5 dpi
uninjured
control
GFP
Rubicon f04462
OK371>UAS-mCD8::GFP
remaining axon debris
5 dpi
p=0.0012
normalized integrated density
30
20
10
0
control
Rubicon f04462

Supplementary Figure 9. Additional Vps34 complex data

a Single-slice images of uninjured wing L1 vein glia expressing *repo-Gal4*-driven *UAS-GFP-ref(2)P* and co-expressing the indicated RNAi-s. Scale bar: 5 µm. **b** Quantification of the number of GFP-Ref(2)P puncta in single-slice images of genotypes in **a**. Statistical analysis was performed with unpaired, two-tailed Mann-Whitney test (*Atg14*) or t-test (*Vps34*). n=19, 18, 10 and 10 biologically independent animals. *w* RNAi serves as negative control. Independent experiments are separated by a dashed line. **c** Coding sequence change in *Rubicon*^{fs1} *and Rubicon*^{fs2} mutants. Number of remaining amino acids (aa) in the truncated protein products are indicated. **d**, **e** *Rubicon* mRNA levels measured by qRT-PCR in the indicated genotypes. Statistics was performed with unpaired, two-tailed t-test, shown is the mean with standard deviation. n=3 biologically independent RNA samples for all. **f** Single slice images of degenerating wing nerves at 5 dpi in the *Rubicon*^{f04462} mutant and uninjured contralateral wing nerves (left), and quantification of axon debris abundance in single-slice images (right). Unpaired, two-tailed Mann-Whitney test was used for statistics. n=14 and 17 biologically independent animals. Scale bar: 5 µm. Truncated violin plots with median and quartiles are shown. Source data are provided as a Source Data file.

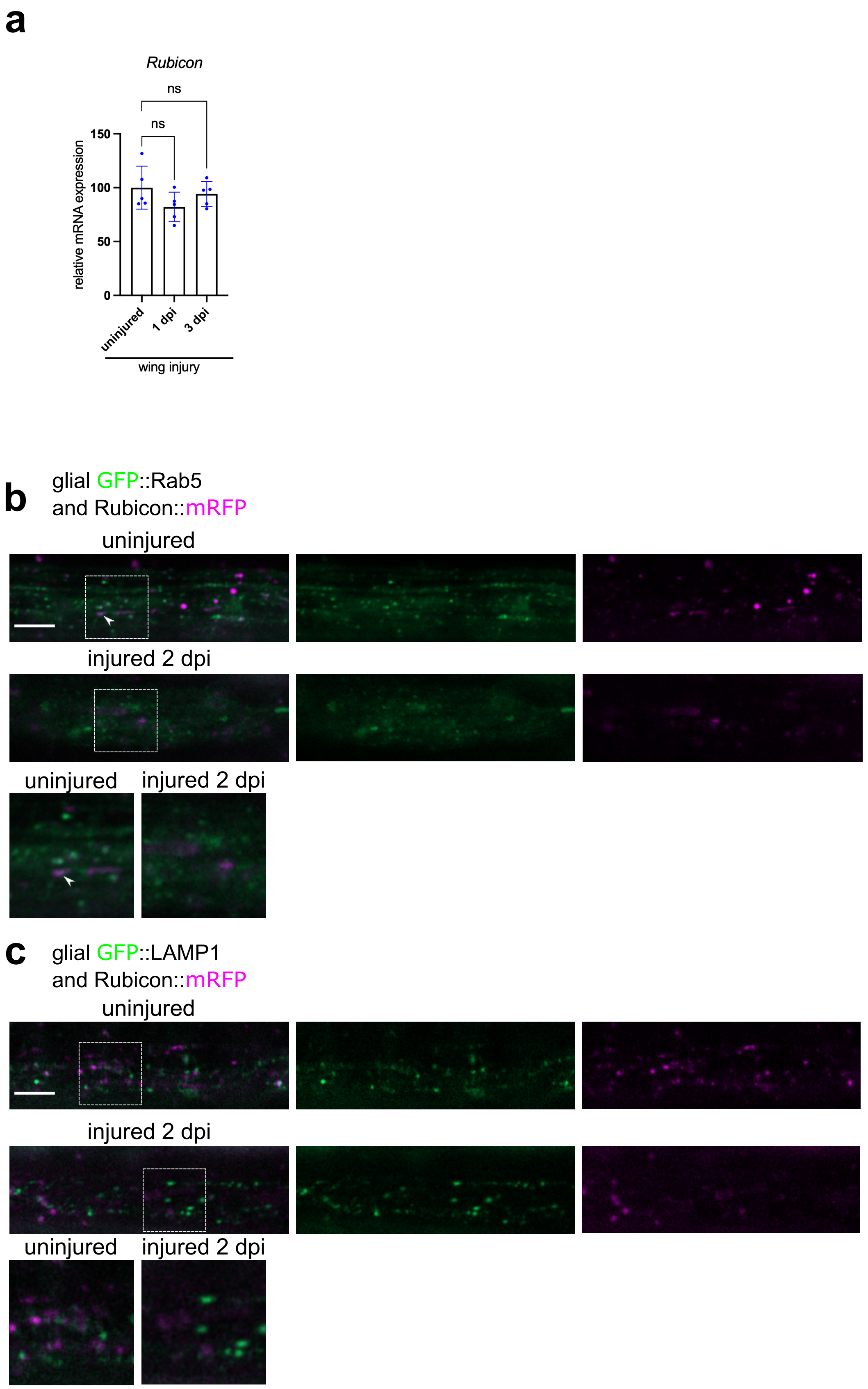
a
Rubicon
ns
ns
relative mRNA expression
150
100
50
0
uninjured
1 dpi
3 dpi
wing injury
b
glial GFP::Rab5
and Rubicon::mRFP
uninjured
injured 2 dpi
uninjured
injured 2 dpi
c
glial GFP::LAMP1
and Rubicon::mRFP
uninjured
injured 2 dpi
uninjured
injured 2 dpi

Supplementary Figure 10. *Rubicon* expression does not change at the transcript level after injury and Rubicon scarcely colocalizes with Rab5 or LAMP1 in glia

a *Rubicon* transcript levels measured by qRT-PCR from intact or transected wings collected at the indicated time points. One-way ANOVA with Šídák's multiple comparisons test, p values were corrected for multiple comparisons. p=0.0916 and 0.5622, ns - not significant. Shown is the mean with standard deviation. n=5 biologically independent RNA samples. **b**, **c** Confocal single-slice images of uninjured and injured wing nerves at 2 dpi expressing Rubicon::mRFP1 and GFP::Rab5 in glia (*repo-Gal4> UAS-Rubicon::mRFP1, UAS-GFP::Rab5*) (**b**) or Rubicon::mRFP1 and GFP::LAMP1 (*repo-Gal4> UAS-Rubicon::mRFP1, >UAS-GFP::LAMP1*) (**c**). Magnified images of the areas outlined by the dashed rectangles are shown below. Arrowhead points to co-localizing puncta. Scale bar: 5 µm. Experiments were independently repeated twice with similar results. Source data are provided as a Source Data file.

**Supplementary Table 1.**	**qPCR primer pair sequences used in this study**
	Atg5 qPCR fwd: 5’ AGAGCGGTTGGATGTCGCA
	Atg5 qPCR rev: 5’ CACGCGAACTGGGCAAAACA
	Atg16 qPCR fwd: 5’ GATCGCTTGTGGATCAGCGG
	Atg16 qPCR rev: 5’ GTTTACGGCGGTGCTGTGT
	Syx17 qPCR fwd: GCATGAAGGAATTTGCGGAGC
	Syx17 qPCR rev: GTGTGCGGGTAGACTGTTCA
	rubicon fwd: AGCACAAGGAACTGGCGAAGG
	rubicon rev: ATTGAAGAATGACTGCTCCCTCGTTG
	UVRAG qPCR fwd: CGTCAACTCAACGCATTTGCC
	UVRAG qPCR rev: ATAGTTATGCTCCAGTCGCGG
	Vps34 qPCR fwd: GTGCTAGAGAACGGCAACTTC
	Vps34 qPCR rev: CACCAGTTTCACCAGTTTGACC
	Atg14 qPCR fwd: CGGAGTCAGAGGACGAAAAC
	Atg14 qPCR rev: CGATGGTAGACTGCTGGTTG
	Atg13 qPCR fwd: AGTGGTGGGCTCCAAATGAG
	Atg13 qPCR rev: GTTTGTCCCTGCCTCTCTCC
	Atg17 qPCR fwd: TCTTAACAAGGACAAGTGCCG
	Atg17 qPCR rev: GATCGTAGGCAGAGCTTCTCA
	$RpL32^{FWD}$ 5’ TGCTAAGCTGTCGCACAAATGGC 3’
	$RpL32^{REV}$ 5’ CGATCCGTAACCGATGTTGGGC 3’;
