# Supplementary material for: LC3-associated phagocytosis promotes glial degradation of axon debris after injury in Drosophila models
Source: Nat Commun. 2023 May 29;14:3077. doi: 10.1038/s41467-023-38755-4 (PMC10227080; doi:10.1038/s41467-023-38755-4)
Supplement: Supplementary file 3 — Reporting Summary [file 41467_2023_38755_MOESM3_ESM.pdf]

nature portfolio

Corresponding author(s): Aron Szabo
Gabor Juhasz

Last updated by author(s): Apr 20, 2023

Reporting Summary

Nature Portfolio wishes to improve the reproducibility of the work that we publish. This form provides structure for consistency and transparency in reporting. For further information on Nature Portfolio policies, see our Editorial Policies and the Editorial Policy Checklist.

Statistics

For all statistical analyses, confirm that the following items are present in the figure legend, table legend, main text, or Methods section.

n/a	Confirmed	
☐	☒	The exact sample size (*n*) for each experimental group/condition, given as a discrete number and unit of measurement
☐	☒	A statement on whether measurements were taken from distinct samples or whether the same sample was measured repeatedly
☐	☒	The statistical test(s) used AND whether they are one- or two-sided<br>*Only common tests should be described solely by name; describe more complex techniques in the Methods section.*
☒	☐	A description of all covariates tested
☐	☒	A description of any assumptions or corrections, such as tests of normality and adjustment for multiple comparisons
☐	☒	A full description of the statistical parameters including central tendency (e.g. means) or other basic estimates (e.g. regression coefficient) AND variation (e.g. standard deviation) or associated estimates of uncertainty (e.g. confidence intervals)
☐	☒	For null hypothesis testing, the test statistic (e.g. F, t, r) with confidence intervals, effect sizes, degrees of freedom and P value noted<br>*Give P values as exact values whenever suitable.*
☒	☐	For Bayesian analysis, information on the choice of priors and Markov chain Monte Carlo settings
☒	☐	For hierarchical and complex designs, identification of the appropriate level for tests and full reporting of outcomes
☒	☐	Estimates of effect sizes (e.g. Cohen's d, Pearson's r), indicating how they were calculated

Our web collection on statistics for biologists contains articles on many of the points above.

Software and code

Policy information about availability of computer code

Data collection	Microscopy image acquisition was performed in ZEN 2.3 (Zeiss), qPCR experiments were measured and analysed with Rotor-Gene software 2.3.1.49 (Qiagen) .
Data analysis	For image analysis, Fiji v2.9.0/1.53t (open source software) was used, statistical analysis was performed with Prism 9.5.1 (GraphPad). Primers were designed with Primer-BLAST (https://www.ncbi.nlm.nih.gov/tools/primer-blast).

For manuscripts utilizing custom algorithms or software that are central to the research but not yet described in published literature, software must be made available to editors and reviewers. We strongly encourage code deposition in a community repository (e.g. GitHub). See the Nature Portfolio guidelines for submitting code & software for further information.

Data

Policy information about availability of data

All manuscripts must include a data availability statement. This statement should provide the following information, where applicable:

- Accession codes, unique identifiers, or web links for publicly available datasets
- A description of any restrictions on data availability
- For clinical datasets or third party data, please ensure that the statement adheres to our policy

All data needed to evaluate the conclusions in this paper are present in the paper, its Supplementary Information and Source Data. The underlying data for Protein Data Bank structure 4NAW of ATG12~ATG5-ATG16N are available at https://www.rcsb.org/structure/4naw.

Human research participants

Policy information about studies involving human research participants and Sex and Gender in Research.

Reporting on sex and gender	N/A
Population characteristics	N/A
Recruitment	N/A
Ethics oversight	N/A

Note that full information on the approval of the study protocol must also be provided in the manuscript.

Field-specific reporting

Please select the one below that is the best fit for your research. If you are not sure, read the appropriate sections before making your selection.

☒ Life sciences ☐ Behavioural & social sciences ☐ Ecological, evolutionary & environmental sciences

For a reference copy of the document with all sections, see nature.com/documents/nr-reporting-summary-flat.pdf

Life sciences study design

All studies must disclose on these points even when the disclosure is negative.

Sample size

Sample size was not predetermined by statistical methods but was similar as in other publications describing axon debris engulfment and autophagy, aligning with the standards of the field, please see references below. Loss-of-function effects of drpr and various autophagy-related genes on different biological processes were taken as a proxy to estimate effect sizes based on mean or median difference of populations. Sample sizes which gave sufficiently large effect sizes in those assays were used in our study. Information on sample size, independent replicates, statistical methods and descriptors are found in the Figure Legends, Methods and Supplementary Information.

1. Lu, T.-Y., Doherty, J. & Freeman, M. R. DRK/DOS/SOS converge with Crk/Mbc/dCed-12 to activate Rac1 during glial engulfment of axonal debris. Proc. Natl. Acad. Sci. U.S.A. 111, 12544–12549 (2014).
2. Ziegenfuss, J. S., Doherty, J. & Freeman, M. R. Distinct molecular pathways mediate glial activation and engulfment of axonal debris after axotomy. Nat Neurosci 15, 979–987 (2012).
3. Jipa, A. et al. Analysis of Drosophila Atg8 proteins reveals multiple lipidation-independent roles. Autophagy 17, 2565–2575 (2021).
4. Pircs, K. et al. Advantages and Limitations of Different p62-Based Assays for Estimating Autophagic Activity in Drosophila. PLoS One 7, e44214 (2012).
5. Takáts, S. et al. Autophagosomal Syntaxin17-dependent lysosomal degradation maintains neuronal function in Drosophila. J Cell Biol 201, 531–539 (2013).

Data exclusions

No data were excluded from the analyses.

Replication

Experiments were repeated twice and used at least 3 independent biological replicates except for western blots where n=2. All attempts at replication were successful.

Randomization

Drosophila samples were randomly chosen from progeny of crosses and allocated to experimental groups based on genotypes, injury status and days post injury. Covariates were not tested.

Blinding

During image analysis, the evaluator was blinded to the identity (genotype, condition) of the image files. qPCR results were not assessed with blinding due to less possibility for unconscious bias. qPCR provides numerical readouts , uses technical replicates that control for noise and the setup of reactions is highly controlled. Expression values of the target gene are normalized to the expression value of a houskeeping gene. This allows for less chance for bias in experimental setup, data analysis and interpretation. Similarly, Western blot samples were not blinded. This is due to the necessity to load the samples for side-by-side comparison rather in a specified order and not randomly. Bias is reduced by

using similar concentrations of total protein, normalizing test protein band intensities to a houskeeping protein (in our case, tubulin) band intensity and by only comparing samples on the same membrane. There are also no or less regional differences in signal quality or strength to be considered in a Western blot or qPCR experiment as opposed to fluorescence imaging of tissues that justifies blinding of image analysis rather than molecular analysis. Survival analysis of brain injured flies was not blinded as the readout is the number of fly death events in every 2 or 3 days while flies of all conditions are handled identically . This is a well -defined readout not prone to bias.

Reporting for specific materials, systems and methods

We require information from authors about some types of materials, experimental systems and methods used in many studies. Here, indicate whether each material, system or method listed is relevant to your study. If you are not sure if a list item applies to your research, read the appropriate section before selecting a response.

Materials & experimental systems

n/a	Involved in the study
☐	☒ Antibodies
☒	☐ Eukaryotic cell lines
☒	☐ Palaeontology and archaeology
☐	☒ Animals and other organisms
☒	☐ Clinical data
☒	☐ Dual use research of concern

Methods

n/a	Involved in the study
☒	☐ ChIP-seq
☒	☐ Flow cytometry
☒	☐ MRI-based neuroimaging

Antibodies

Antibodies used

anti-GABARAP/GABARAPL1/GABARAPL2 rabbit monoclonal (Abcam ab109364) Clone number EPR4805
anti-HA rabbit polyclonal (Sigma-Aldrich H6908)
anti-alpha-tubulin mouse monoclonal (Developmental Studies Hybridoma Bank AA4.3)
anti-Drpr 8A1 mouse monoclonal, Developmental Studies Hybridoma Bank
anti-GFP rabbit polyclonal, Thermo Fisher Scientific A-11122
goat anti-mouse IRDye 800CW (LI-COR, 926-32210)
goat anti-rabbit IRDye 680RD (LI-COR, 926-68071)
goat anti-rabbit Alexa Fluor 488, A-11034 Thermo Fisher Scientific
goat anti-mouse Alexa Fluor 568, A-11031 Thermo Fisher Scientific

Validation

ab109364: abreview EPR4805, Fig. S3c this study, Jipa et al., 2021, Autophagy, 17, 2565–2575.
H6908: Henn el al., 2007, Journal of Neuroscience 27 (8) 1868-1878
AA4.3: Lőrincz et al. eLife 2019;8:e45631
8A1: Musashe et al., Cell reports 16.7 (2016 Aug 16): 1838-50.
A-11122 : https://www.thermofisher.com/antibody/product/GFP-Antibody-Polyclonal/A-11122, antibody testing data

Animals and other research organisms

Policy information about studies involving animals; ARRIVE guidelines recommended for reporting animal research, and Sex and Gender in Research

Laboratory animals

Our studies used Drosophila melanogaster, an insect species for which no ethical approval is required according to Biological Research Center, Szeged regulations. Flies were maintained at 25°C on a cornmeal-yeast-agar-dextrose medium with Nipagin as preservative. Drosophila melanogaster adult males 4-8 days after eclosion were used for the experiments except when stated otherwise. We preferred males over females due to long incubation times of injured animals during which female egg laying and resulting larval stirring of fly food would lead to enhanced mortality of adults as they stick to fly food. 4-8 days old females were only used in experiments where trans-heterozygous combinations of X chromosomal mutations were studied, including Fig. 1d, S1b: Atg55cc5/ Atg5CR00038 and Figure 6g, S2: Rubiconfs1/Rubiconfs2.
CRISPR-Cas9 genome-edited animals created in this study: Rubicon(fs) mutants. Transgenic animals generated by P-element based transformation in this study: pTWR-dRubicon, genomic promoter-driven Atg5–3xHA.
The following, already existing stocks were used:
OK371-Gal4, UAS-mCD8-GFP
UAS-GFP-LAMP1
Atg8aΔ4
Atg16Δ67
Atg16Δ129
Atg55cc5
Atg8aG116*
Atg17/FIP200Δ130
UAS-GFP-ref (2)P
Atg16-3xHA
3xmCherry-Atg8a
Atg101d6h

UAS-WT dRubicon::HA
repo-GeneSwitch
repo-Gal4
drprd5
Mi{Trojan-QF2.2}VGlutMI04979-TQF2.2
QUAS-mCD8::GFP
Atg17/FIP200MI01469
Atg16MI00187
Rubiconf04462
Atg5CR00038
wGL00094
Atg14HMS02025
UVRAGHMS01357
Atg5JF02703
Atg5HMS01244
Atg1GL00047
Syx17JF01937
Atg8aJF02895
w sgRNA TKO.GS02468
Rubicon sgRNA TKO.GS04756
UASp-mCherry-Atg8a
nSyb-lexA.DBD::QF.AD, 13x lexAop2-IVS-myr::GFP
13xLexAop2-CD4::tdTomato (77139), UAS-IVS-myr::tdTomato
UAS-Rab7::GFP
UAS-GFP::Rab5
tubP-Gal4
and 10XQUAS-6XGFP; Orco-2A-QF2
Atg8aKK102155
RubiconKK108247
Vps34KK107602
Atg13KK100340
Atg17/FIP200KK101847
Atg16GD10140
Atg16KK102326

Wild animals	No wild animals were used.
Reporting on sex	Drosophila melanogaster males 4-8 days after eclosion were used for the experiments except when stated otherwise. We preferred males over females due to long incubation times of injured animals during which female egg laying and resulting larval stirring of fly food would lead to enhanced mortality of adults as they stick to fly food. 4-8 days old females were only used in experiments where trans-heterozygous combinations of X chromosomal mutations were studied, including Fig. 1d, S1b: Atg55cc5/ Atg5CR00038 and Figure 6g, S2: Rubiconfs1/Rubiconfs2.
Field-collected samples	No field-collected samples were involved in this study.
Ethics oversight	No ethical approval was required for Drosophila melanogaster experiments according to Biological Research Center, Szeged regulations.

Note that full information on the approval of the study protocol must also be provided in the manuscript.
